# Ponatinib-induced psoriasis treated with tildrakizumab

**DOI:** 10.1016/j.jdcr.2025.04.038

**Published:** 2025-05-28

**Authors:** Kyle Mueller, Alicia Goldenberg, Drew Kuraitis

**Affiliations:** a Jacobs School of Medicine and Biomedical Sciences, University at Buffalo, Buffalo, New York; b Department of Dermatology, Roswell Park Comprehensive Cancer Center, Buffalo, New York; c Department of Pathology and Laboratory Medicine, Roswell Park Comprehensive Cancer Center, Buffalo, New York; d Department of Dermatology, Tulane University, New Orleans, Louisiana

**Keywords:** adverse event, cutaneous toxicity, interleukin-23, leukemia, ponatinib, psoriasis, tildrakizumab, tyrosine kinase inhibitor

## Introduction

Ponatinib is a tyrosine kinase inhibitor (TKI) used in treating Philadelphia chromosome-positive (Ph^+^) leukemia. Ponatinib can induce a variety of cutaneous toxicities; however, as a newer iteration of TKI, ponatinib’s cutaneous toxicity profile may not be fully realized at this time. Herein, we report a psoriasiform eruption following treatment with ponatinib, an adverse event not yet seen with this drug and rarely reported with older TKIs imatinib and nilotinib. The eruption was managed using anti-interleukin (IL) 23 antibody tildrakizumab, a novel use of a biologic medication for TKI-induced psoriasis that allowed the patient to continue receiving ponatinib therapy for B-cell acute lymphoblastic leukemia.

## Case presentation

**Fig 1:**
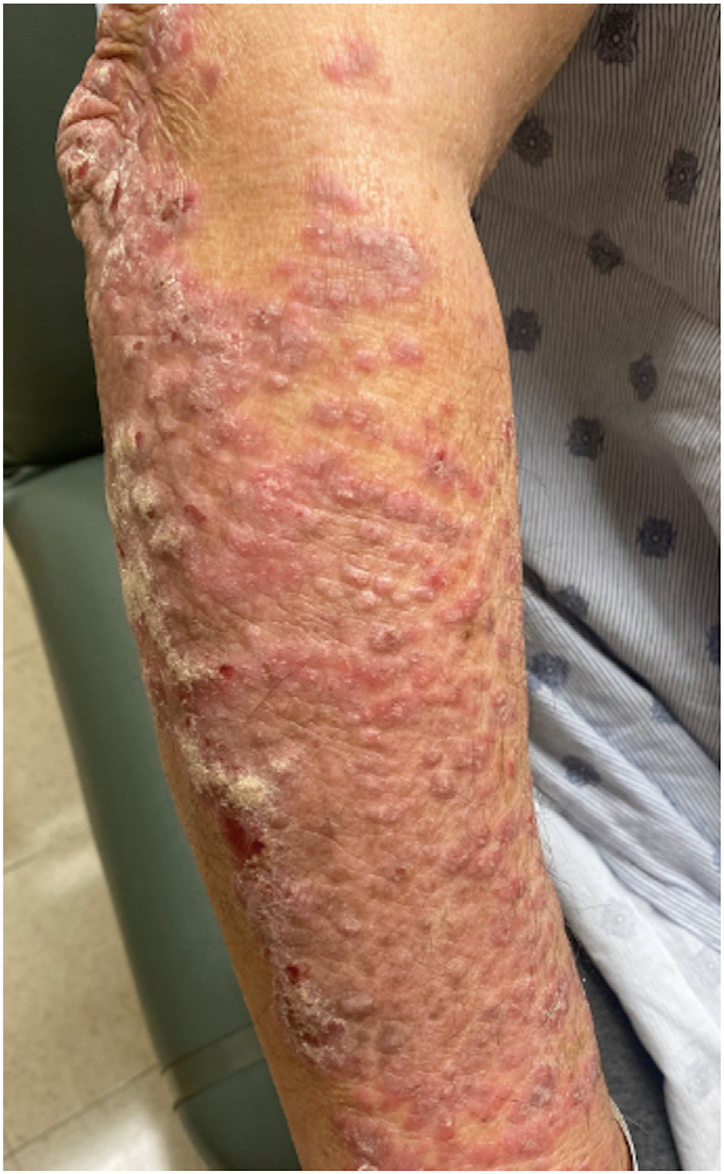
Initial presentation of ponatinib-induced psoriasis. Coalescing erythematous indurated scaling papules and plaques to the right forearm, which started shortly after ponatinib initiation.

**Fig 2:**
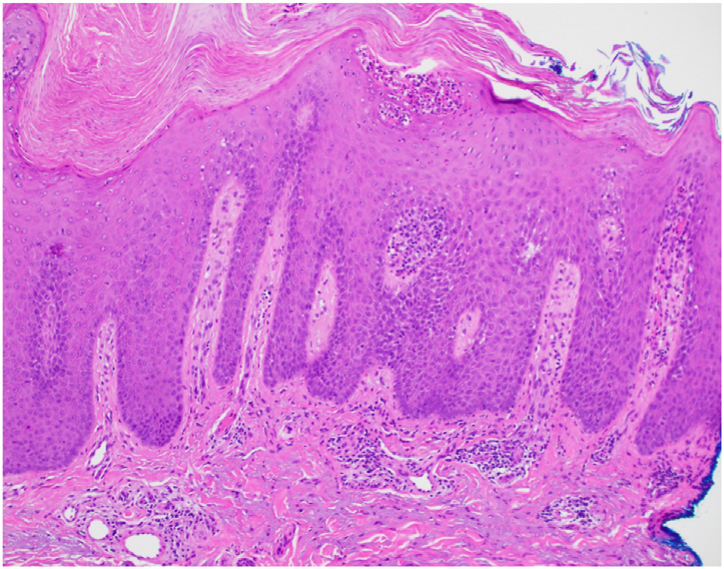
Histological findings of ponatinib-induced psoriasis. Biopsy demonstrates epidermal acanthosis with overlying parakeratosis and collections of neutrophils within the stratum corneum. A diminished to absent granular layer is observed. There is a superficial perivascular lymphocytic infiltrate with eosinophils.

**Fig 3:**
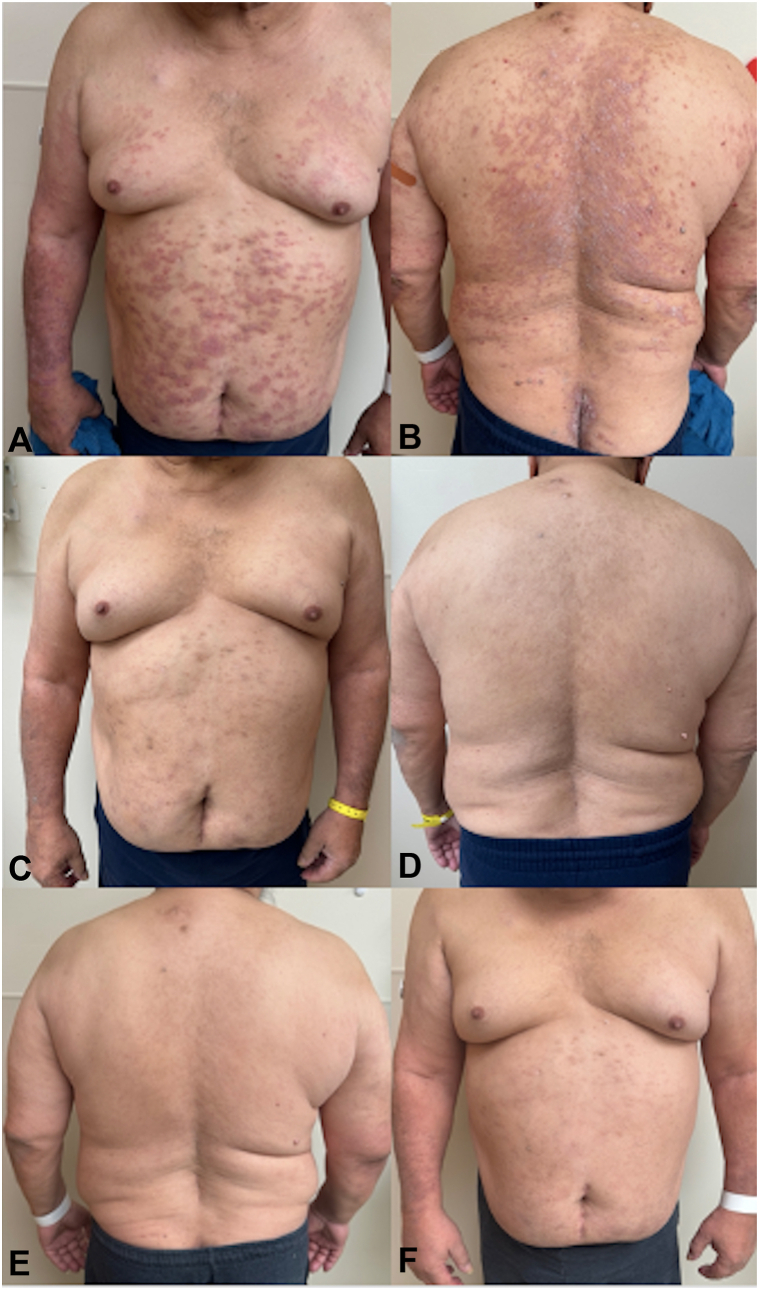
**A, B,** Ponatinib-induced psoriasis to the trunk and arms prior to starting tildrakizumab, **C, D,** with clearance 14 weeks after only receiving the first loading dose. **E, F,** Sustained clearance 12 weeks after resuming tildrakizumab.

A 66-year-old man with B-cell acute lymphoblastic leukemia presented to the dermatology clinic for evaluation of a rash. 14 months prior he started ponatinib and after a few months he developed an intensely pruritic rash most prominent over his arms, with coalescing erythematous scaly papules and plaques (Fig 1). He never had a similar rash before and denied a personal or family history of psoriasis. Biopsy of the right arm demonstrated epidermal acanthosis with overlying parakeratosis, collections of neutrophils within the stratum corneum, and presence of eosinophils, consistent with psoriasiform drug eruption (Fig 2). Two weeks later, he noted improvement in pruritus and lesion intensity with triamcinolone 0.1% cream, and he was not developing new lesions. He did not return to dermatology for follow-up until 8 months later, when he presented with expanding psoriasiform plaques to the trunk, extremities, and gluteal cleft, with approximately 30% body surface area involvement (Fig 3, *A* and *B*). During this time, he had continued with as-needed use of triamcinolone 0.1%. Due to extensive pruritus and body surface area, disease control could not be maintained with topical therapies alone, and he was started on standard dosing of tildrakizumab with 100 mg at week 0. He did not return for his week 4 tildrakizumab, reportedly because his skin cleared within 1 week. 14 weeks later, he again returned to clinic because his rash was starting to recur on the forearms, although his trunk still remained clear (Fig 3, *C* and *D*). The decision was made to continue with maintenance dosing every 12 weeks instead of repeating a loading dose, and he received another 100 mg of tildrakizumab. Twelve weeks later, he returned for tildrakizumab administration and remained clear (Fig 3, *E* and *F*), with plans to continue standard maintenance to manage cutaneous toxicity of ponatinib. During each of his visits, screening for psoriatic arthritis was unremarkable, and he had no significant nail findings.

## Discussion

Ponatinib, a third-generation TKI, targets the breakpoint cluster region (BCR) and Abelson (ABL) fusion protein, known as the oncoprotein BCR-ABL, in the management of Ph^+^ leukemia, including Ph^+^ B-cell acute lymphoblastic leukemia and chronic myeloid leukemia.^1^ As the third generation of TKIs against BCR-ABL, ponatinib can overcome the BCR-ABL kinase domain mutation that contributes to first-generation and second-generation TKI evasion.^1^ Off-target effects are common, as ponatinib, along with prior-generation TKIs, can bind the tyrosine kinase domain of other proteins involved in intracellular signaling.^1^ Possibly consequent to various downstream effects and dysregulation of inflammation, ponatinib can induce cutaneous toxicities, known to include panniculitis, pityriasis rubra pilaris–like eruptions, ichthyosis-like eruptions, and maculopapular rash.^1^

To the best of our knowledge, this case constitutes the first reported psoriasiform eruption following ponatinib therapy; however, older TKIs imatinib and nilotinib have reportedly caused similar psoriatic eruptions.^2^ Given the current spectrum of ponatinib’s known cutaneous toxicity, induction of psoriasis is a reasonable addition. Pityriasis rubra pilaris shares an immunogenic pathway with psoriasis via phospholipase A2 enzymes,^3^ while ichthyosis pathogenesis resembles psoriasis with IL-23/Th17-skewing in skin.^4^ Furthermore, the induction of psoriasis by imatinib and nilotinib implies a related mechanism by ponatinib. The specificity of these drugs to BCR-ABL and other similar targets distinguishes these inhibitors from other TKIs. This is an important distinction, as newer agents, such as inhibitors of tyrosine kinase 2 of the Janus kinase family, can be efficacious in the treatment of psoriasis.^5^

Given that anti-IL-23 monoclonal antibody tildrakizumab cleared this patient’s psoriasis, the IL-23/Th17 pathway is likely implicated in this patient’s cutaneous toxicity. Development of psoriasis via IL-23 is dependent on P2X7 receptor signaling and subsequent activation of NOD-like receptor P3 (NLRP3) in cutaneous myeloid dendritic cells.^6^ Furthermore, activity of NLRP3 predisposes patients to psoriatic phenotype.^6^ Studies have shown that TKIs, especially imatinib in the treatment of chronic myeloid leukemia, through off-target effects, activate NLRP3 specifically in primary myeloid cells.^7^ This off-target activation of NRLP3 likely contributes to ponatinib-induced psoriasis in Ph^+^ leukemia treatment given ponatinib’s structural similarity to imatinib.^1^ According to this model, tildrakizumab would inhibit IL-23 signaling and reduce total activation of NLRP3, providing a possible explanation for the therapeutic benefits seen in our patient. Regardless of the mechanism, tildrakizumab was highly effective in management. Additionally, tildrakizumab’s anti-IL-23/Th17 effects may have a benefit in ponatinib therapy, as IL-17A activates BCR-ABL signaling, supporting Ph^+^ leukemia progression.^8^

Standard tildrakizumab dosing includes loading doses at weeks 0 and 4, followed by dosing every 12 weeks, reaching steady-state concentrations at 16 weeks.^9^ With the antibody half-life being 23 days, 55% to 58% of patients in the main clinical trial reSURFACE 1 achieved clearance or minimal psoriasis at 12 weeks.^9^ Thus, the patient’s reportedly sufficient clearance by 4 weeks constitutes a rapid response. Additionally, the patient’s response remained sufficient up to 14 weeks after receiving only the week 0 dose. To date, studies have not investigated the benefit of a single dose, but in reSURFACE 1, 54% of patients who received 100 mg doses only at weeks 0, 4, and 16 experienced relapsing psoriasis prior to week 64, with a median time to relapse of 34 weeks.^10^ Regardless of the nonstandard treatment course, tildrakizumab therapy was efficacious and enabled uninterrupted ponatinib therapy for our patient’s malignancy. To the best of our knowledge, this case constitutes the first reported use of a biologic to manage TKI-induced psoriasis.

In this report, we present a case of ponatinib-induced psoriasis, adding to the drug’s profile of potential cutaneous toxicities. It is possible that off-target effects of ponatinib activate NRLP3 to drive an IL-23/Th17-mediated process, leading to the development of psoriasis. Treatment with tildrakizumab led to rapid and sustained clearance.

## Conflicts of interest

None disclosed.
